# Milk microbiome diversity and bacterial group prevalence in a comparison between healthy Holstein Friesian and Rendena cows

**DOI:** 10.1371/journal.pone.0205054

**Published:** 2018-10-24

**Authors:** Paola Cremonesi, Camilla Ceccarani, Giulio Curone, Marco Severgnini, Claudia Pollera, Valerio Bronzo, Federica Riva, Maria Filippa Addis, Joel Filipe, Massimo Amadori, Erminio Trevisi, Daniele Vigo, Paolo Moroni, Bianca Castiglioni

**Affiliations:** 1 Institute of Agricultural Biology and Biotechnology, National Research Council (CNR), Lodi, Italy; 2 Institute of Biomedical Technologies, National Research Council, (CNR), Segrate, Milan, Italy; 3 Dipartimento di Scienze della Salute, San Paolo Hospital Medical School, Università degli Studi di Milano, Milan, Italy; 4 Dipartimento di Medicina Veterinaria, Università degli Studi di Milano, Milan, Italy; 5 Laboratory of Cellular Immunology, Istituto Zooprofilattico Sperimentale della Lombardia e dell'Emilia-Romagna, Brescia, Italy; 6 Department of Animal Sciences, Food and Nutrition (DIANA), Facoltà di Scienze Agrarie, Alimentari ed Ambientali, Università Cattolica del Sacro Cuore, Piacenza, Italy; 7 Quality Milk Production Services, Animal Health Diagnostic Center, Cornell University, Ithaca, NY, United States of America; University of Illinois, UNITED STATES

## Abstract

Dry and early lactation periods represent the most critical phases for udder health in cattle, especially in highly productive breeds, such as the Holstein Friesian (HF). On the other hand, some autochthonous cattle breeds, such as the Rendena (REN), have a lower prevalence of mastitis and other transition-related diseases. In this study, milk microbiota of 6 HF and 3 REN cows, all raised on the same farm under the same conditions, was compared. A special focus was placed on the transition period to define bacterial groups’ prevalence with a plausible effect on mammary gland health. Four time points (dry-off, 1 d, 7–10 d and 30 d after calving) were considered. Through 16S rRNA sequencing, we characterized the microbiota composition for 117 out of the 144 milk samples initially collected, keeping only the healthy quarters, in order to focus on physiological microbiome changes and avoid shifts due to suspected diseases. Microbial populations were very different in the two breeds along all the time points, with REN milk showing a significantly lower microbial biodiversity. The taxonomic profiles of both cosmopolitan and local breeds were dominated by Firmicutes, mostly represented by the *Streptococcus* genus, although in very different proportions (HF 27.5%, REN 68.6%). Large differences in HF and REN cows were, also, evident from the metabolic predictive analysis from microbiome data. Finally, only HF milk displayed significant changes in the microbial composition along the transition period, while REN maintained a more stable microbiota. In conclusion, in addition to the influence on the final characteristics of dairy products obtained from milk of the two breeds, differences in the milk microbiome might, also, have an impact on their mammary gland health.

## Introduction

The complex variety of microbes inhabiting living animals and the reciprocal interactions they entertain among themselves and with their hosts have been increasingly pointed out by the evolution of molecular and “-omics” technologies [1]. Among these new technologies, metagenomics enables the characterization of a microbial population in a culture-independent manner [2], providing a powerful tool for identifying dominant and subdominant microbes and their dynamics in highly complex ecosystems.

On their skin, in gut, oro-pharyngeal, urinary, and genital tracts, all animals host a broad diversity of microbial communities that, through intricate mutualistic interactions, have evolved with them and play crucial roles in their biology and health [3]. Until recently, the mammary gland, which was considered as a sterile organ, has also been included among these organ systems displaying a unique microbiota [1], although the extent and origin of microbial colonization is still under debate [4]. According to the current scientific literature, direct or indirect contact with milk are the two major origins of its microbiota composition. Direct contact is associated with the microbial ecosystems involving the animal's teat canal and surface status and contact with milking machines or other dairy equipment. Indirect contact concerns various environmental elements, such as bedding material, feces, forage, drinking and washing water, parlor air (stable and milking) and the milker [5–6]. Several studies support the hypothesis that bacteria occur in milk not only as the result of external colonization, as bacterial isolates present in the mammary gland have been observed to be genotypically different from the same species found on skin within the same host [1]. Various authors described the ability of some microbes to move from intestinal lumen to the mammary gland through an entero-mammary pathway [1,7].

Most studies on the dairy ruminant milk microbiota have focused on how the microbial composition changes during food processing and on its impact on milk quality, product maturation, flavor, taste, texture development and product shelf life [8–9]. Other studies have investigated the impact of different dairy cattle diets on milk microbial communities [10], how milk microbiota changes during mastitis or following antimicrobial treatments, and the effects on milk microbiota of different therapy conditions during the dry period [11–13]. The dry period and the early lactation period represent the most critical phases for udder health [14–15]. Indeed, during the peripartum period, dysregulations of the immune system can justify the onset of many metabolic and infectious diseases in dairy cows and could also have a role in the variability of the microbial mammary gland colonization [16]. The highest incidence of new intra-mammary infections (IMI) is usually recorded in highly productive, selected dairy breeds such as the Holstein Friesian (HF) in the first 2–3 weeks after calving [17]. This partly accounts for the highest culling prevalence routinely observed in the first 2 months of lactation [18]. HF records are in contrast with the low preponderance of clinical mastitis in some autochthonous cows such as the Rendena breed (REN) [19–20]. The Rendena is an indigenous Italian dual-purpose alpine cattle breed with good aptitude to pasture and appreciable milk production (< 5,000 kg of milk per lactation). Animals are mainly reared in Northeastern Italy, especially in low-output systems in which pasture represents the main source of feeding during the summer season. In a previous study, this autochthonous breed was suggested to have a higher resistance to disease in comparison to HF breed reared in the same conditions [20].

The aim of this study was to characterize and compare the milk microbiota of HF and REN cows reared on a single mixed-breed farm under the same management conditions to define bacterial group prevalence with a plausible effect on mammary gland health.

## Materials and methods

### Ethic statement, animals and sampling

This study was conducted in one commercial dairy farm situated in Pavia (Italy) whose owner has established a long-standing and fruitful collaboration with the “Dipartimento di Medicina Veterinaria” at “Università degli Studi”, Milan, Italy. The research protocol was reviewed and approved by Italian Ministry of Health (authorization n. 628/2016-PR) and the methods were carried out in accordance with the approved guidelines. Sampling was conducted on 6 HF and 3 REN cows, kept in a loose housing system during the dry period and after parturition in a tie-stall housing system [20]. The lower number of sampled REN cows was dependent on their availability in the farm. All HF cows were 6 years old, all REN cows 5 years old and all animals were between 2 and 4 lactations (average: 3.6 for HF and 2.7 for REN) with average lactation duration of 340 and 386 days for HF and REN, respectively (range: 257–420 for HF; 302–456 for REN). Milking equipment was evaluated during the study period by the Regional Breeding Association using a complete ISO 6690:2007-defined evaluation (ISO, 2007) to avoid changes in teat dimensions as well as in the teat tissue, such as congestion and hyperkeratosis [21].

During sampling, cows were milked twice daily and were fed *ad libitum* with a silage-free total mixed ration using alfalfa hay, straw and mineral and vitamin-supplemented feed. More details on the characteristic of diets during dry-off and lactating periods are reported in S1 Table. HF cows produced about 42% more milk than REN (average milk yield 5,366 kg *vs*. 3,769 kg for HF and REN, respectively; p = 0.0147), whereas milk fat and protein content (3.52% *vs*. 3.37% and 3.02% *vs*. 3.08% for HF and REN, respectively) were comparable between the two breeds. No dry cow therapy was used, and all cows remained healthy for the period of the study.

### Sample collection

Quarter milk samples were collected at four specific time points: dry-off (T1), 1 d after calving (T2), 7–10 d after calving (T3) and 30 d after calving (T4), as in [20]. Time point T2 corresponds to colostrum sampling. The first streams of foremilk were manually discarded, teat ends were cleaned and approximately 10 ml of milk was collected aseptically from each quarter, into separate vials. Samples were delivered to the laboratory at 4°C, immediately processed for bacteriological analysis and SCC, and frozen at -20°C for metagenomics analysis.

### Bacteriological analysis and SCC

To define udder health, bacteriological analysis and somatic cell counts (SCC) were performed as previously described [20]. Briefly, 10 μl of milk was plated using blood agar plates containing 5% defibrinated bovine blood and incubated aerobically at 37°C with evaluation after 24 and 48 hours. Bacteria were identified according to the guidelines of National Mastitis Council [22]. For each quarter, SCC was determined by an automated fluorescent microscopic somatic cell counter (Bentley Somacount 150, Bentley Instrument, Chaska, MN, USA).

Healthy quarter milk samples were defined as in [20]: (a) for T1 and T2: negative bacteriological culture growth (udder pathogens); (b) for T3 and T4: SCC < 200,000 cells/ml and negative bacteriological culture growth. Since an increase in SCC in dry-off milk and colostrum samples is typically observed [23], no exclusion criteria based on SCC was applied to T1 and T2 samples.

### DNA extraction, library preparation and sequencing

For each quarter, 5 ml of milk sample was centrifuged; DNA was extracted by using a method based on the combination of a chaotropic agent, guanidium thiocyanate, with silica particles, to obtain bacterial cell lysis and nuclease inactivation [24]. The choice of the extraction method was based on previous research [25], considering its good sensitivity and the lack of influence by matrix-derived factors [24]. The method was shown to be suitable for healthy milk samples with a low bacterial load [25] and produced good results in samples extracted from whole milk. DNA quality and quantity were assessed using a NanoDrop ND-1000 spectrophotometer (NanoDrop Technologies, Wilmington, DE, USA). The isolated DNA was stored at -20°C until use.

Bacterial DNA was amplified using the primers described in literature [26] which target the V3-V4 hypervariable regions of the 16S rRNA gene. All PCR amplifications were performed in 25 μl volumes per sample. A total of 12.5 μl of Phusion High-Fidelity Master Mix 2× (ThermoFisher Scientific, Walthem, MA, USA) and 0.2 μl of each primer (100 μM) were added to 2 μl of genomic DNA (5 ng/μl). Blank controls (i.e.: no DNA template added to the reaction) were also performed. A first amplification step was performed in an Applied Biosystem 2700 thermal cycler (ThermoFisher Scientific). Samples were denatured at 98°C for 30 s, followed by 25 cycles with a denaturing step at 98°C for 30 s, annealing at 56°C for 1 min and extension at 72°C for 1 min, with a final extension at 72°C for 7 min. Amplicons were cleaned with Agencourt AMPure XP (Beckman, Coulter Brea, CA, USA) and libraries were prepared following the 16S Metagenomic Sequencing Library Preparation Protocol (Illumina, San Diego, CA, USA). The libraries obtained were quantified by Real Time PCR with KAPA Library Quantification Kits (Kapa Biosystems, Inc., MA, USA), pooled in equimolar proportion and sequenced in one MiSeq (Illumina) run with 2×300-base paired-end reads.

### Microbiota profiling

The reads obtained were analyzed merging pairs using Pandaseq [27] and by discarding low quality reads. Filtered reads were processed using the QIIME pipeline (v 1.8.0) [28], clustered into Operational Taxonomic Units (OTUs) at 97% identity level and taxonomically assigned via RDP classifier [29] against the Greengenes database (release 13_8 http://greengenes.secondgenome.com). Alpha-diversity evaluations were performed using “Chao1” and “observed species” metrics and rarefaction curves were employed to determine whether most of the bacterial diversity had been captured. Statistical evaluation of differences in alpha-diversity was performed by a non-parametric Monte Carlo-based test, using 9,999 random permutations. For beta-diversity, principal coordinates analysis (PCoA) was performed using weighted and unweighted UniFrac distances. “Adonis” function, which performs a partitioning of distance matrices among sources of variation using a permutation test with pseudo-F ratios, of the R package “vegan” [30] was employed to determine statistical separation of the microbiota profiles.

Taxonomic classification of all the bacteria, down to the genus-level, was performed on counts of relative abundance. Species-level characterization was performed by BLAST-aligning all reads belonging to genus *Streptococcus* to a custom reference database consisting of all available reference sequences in NIH-NCBI database (ftp://ftp.ncbi.nlm.nih.gov/genomes/refseq/bacteria/) within this genus and having a finishing status of “contigs”, “scaffolds” or “complete genomes”, for a total of 11,420 strains belonging to 68 species. Potential matches were filtered to retrieve an unequivocal classification for each read. A functional prediction of the bacterial metabolic pathways was performed using PICRUSt software (v 1.0.1) [31] and KEGG pathways database [32]. Differences in functional category profiles between breeds were assessed using Bray-Curtis distance among samples and “adonis” permutation-based test on the experimental labels.

### Statistical analysis

Statistical comparisons were performed using MATLAB software (Natick, MA, USA). For evaluating differences in relative abundances of bacterial groups and functional categories, a Mann-Whitney U-test was performed, excluding a normal distribution of data at every level (Shapiro-Wilk test at 0.99 confidence). Correlation between SCC and relative abundances of microbial taxa was assessed through calculation of the Pearson’s coefficient and of the p-values of the related linear model. Unless otherwise stated, p-values < 0.05 were considered as significant.

## Results

Out of 144 samples collected during the experimental period, 19 were discarded after bacteriological and SCC analyses. Thirteen samples were positive for pathogenic bacteria and 6 had a SCC at either T3 or T4 above 200,000 cell/ml. Only samples from healthy quarters were analyzed to focus on physiological microbiome changes and avoid shifts in diversity due to suspected diseases. From the 125 remaining milk quarter samples, 8 samples were, further, excluded, since their microbiota was almost exclusively constituted by only one (i.e.: *Escherichia* spp.) or few environmental (i.e.: *Pseudomonas* spp.) and opportunistic (i.e.: *Staphylococcus* spp.) microorganisms representing more than 35% of the relative bacterial abundance (S1 Fig). The final number of quarter milk samples analyzed was 117, with 74 and 43 samples for HF and REN, respectively (dropout rate: 22.9% and 10.5% for HF and REN, respectively). The milk microbiota structure of HF and REN cows was characterized by a total of 5,257,683 high-quality reads, with a mean of 44,937 ± 3,315 reads per milk sample at the different time points.

### Comparison between breeds

The first aim of this study was to characterize and compare the general microbial profile of HF and REN healthy milk quarters (i.e.: 117 quarter milk samples selected as above). This was done by separately considering the samples derived from all lactation time points and milk quarter data for the two breeds.

**Fig 1:**
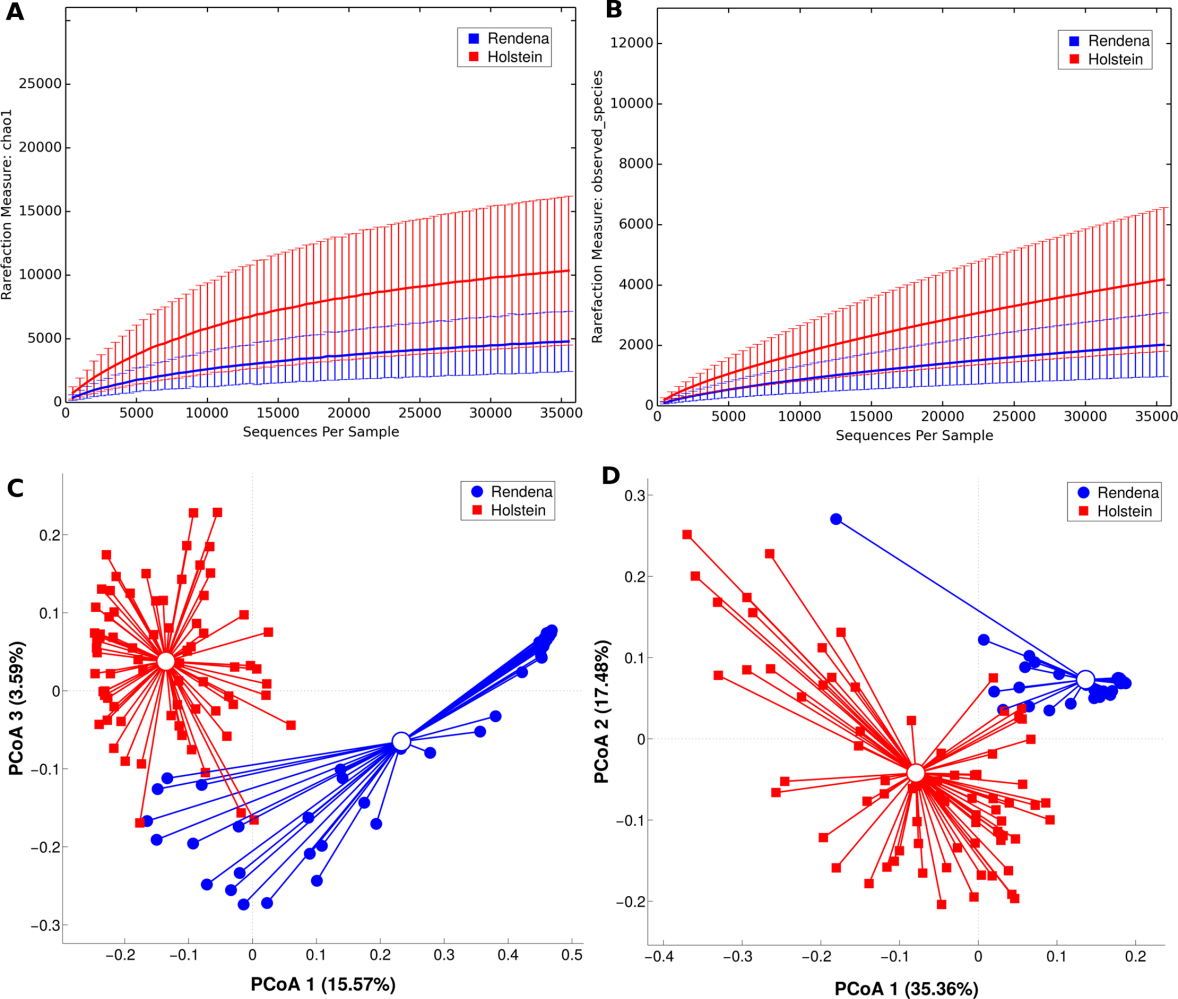
Alpha and beta-diversity among HF (red) and REN (blue). Rarefaction at 35,959 sequences per sample. Alpha-diversity average indexes (plus standard error bars) for phylogenetic diversity Chao1 **(A)** and observed species **(B)** are reported for HF and REN milk samples. Diversity among breeds is statistically significant in all the metrics (including Shannon index, not shown), p-value = 0.001. Beta-diversity analysis is represented by PCoA graphs of weighted UniFrac distance between HF and REN along the principal components **(C-D)**. Each dot represents a single quarter milk sample, while the centroids represent their average value. Separation among the centroids is statistically significant (p-value < 0.001). Percent variance accounted for by the first, second and third principal component is shown along the axis.

As preliminary results, OTU rarefaction curves based on Chao1 and observed species metrics reached the plateau after about 35,000 reads, suggesting that the depth of coverage was sufficient to capture nearly the entire biological diversity within the samples. According to alpha-diversity results, the difference of biodiversity between the two breeds was statistically significant (p-value ≤ 0.01 for both metrics), showing a lower diversity in the microbial ecosystem of REN milk (Fig 1A and 1B). Beta-diversity analysis, on both weighted and unweighted Unifrac distances, showed a pronounced and statistically significant (p-values < 0.001) separation among the breeds as shown in the PCoA graph (Fig 1C and 1D). This revealed major differences in the principal constituents of the microbial community.

**Fig 2:**
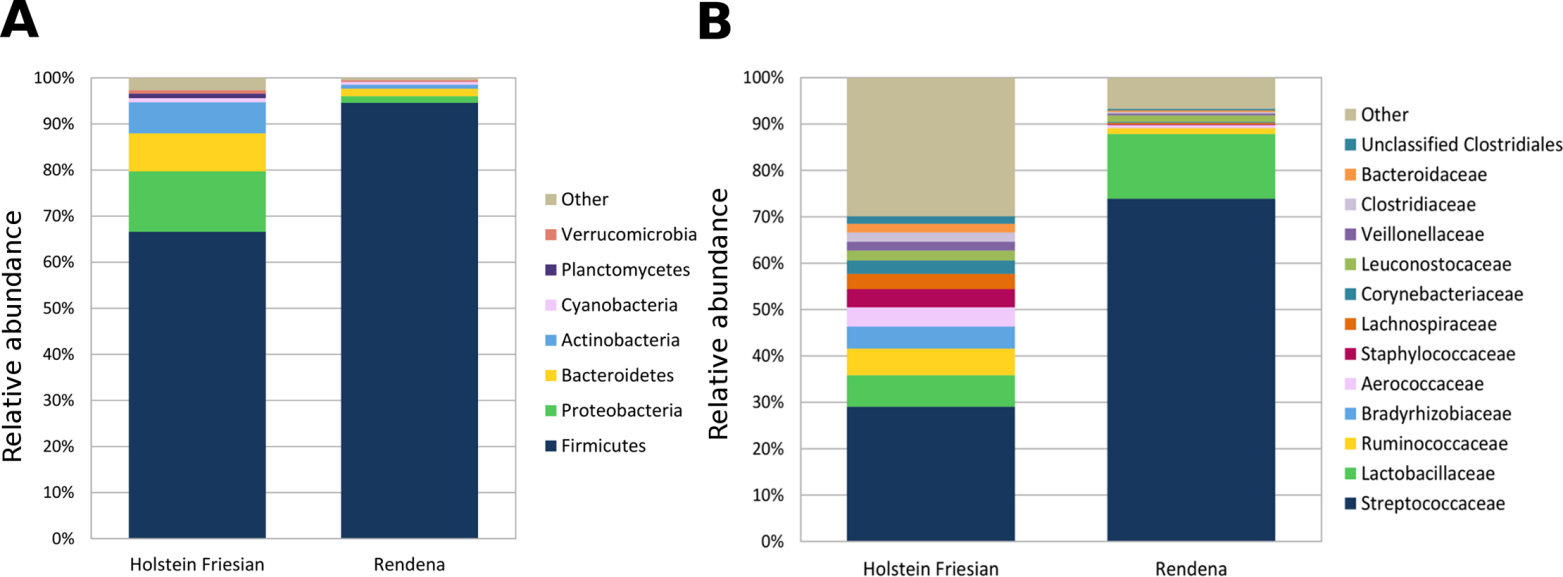
**Distribution of the sequence relative abundances summarized at phylum (A) and family (B) levels.** Relative proportions of bacterial taxonomic groups that were present in at least 1% relative abundance in quarter milk samples at a rarefaction depth of 35,959 sequences. All bacterial taxa present at less than 1% relative abundance were grouped into the “Other” classification.

These data were, further, investigated in terms of the relative abundance in bacterial distribution. In both HF and REN, most of the reads belonged to the phylum Firmicutes, typically the dominant one in dairy cow milk microbiota (Fig 2A). The mean relative abundance of Firmicutes in HF milk was about 66%, as opposed to 94% in REN. HF milk also contained Proteobacteria (<13%), Bacteroidetes (<8%) and Actinobacteria (< 6%), which accounted for only about 1% each of the total relative abundance in REN milk. All these differences were highly significant (p < 0.001).

**Fig 3:**
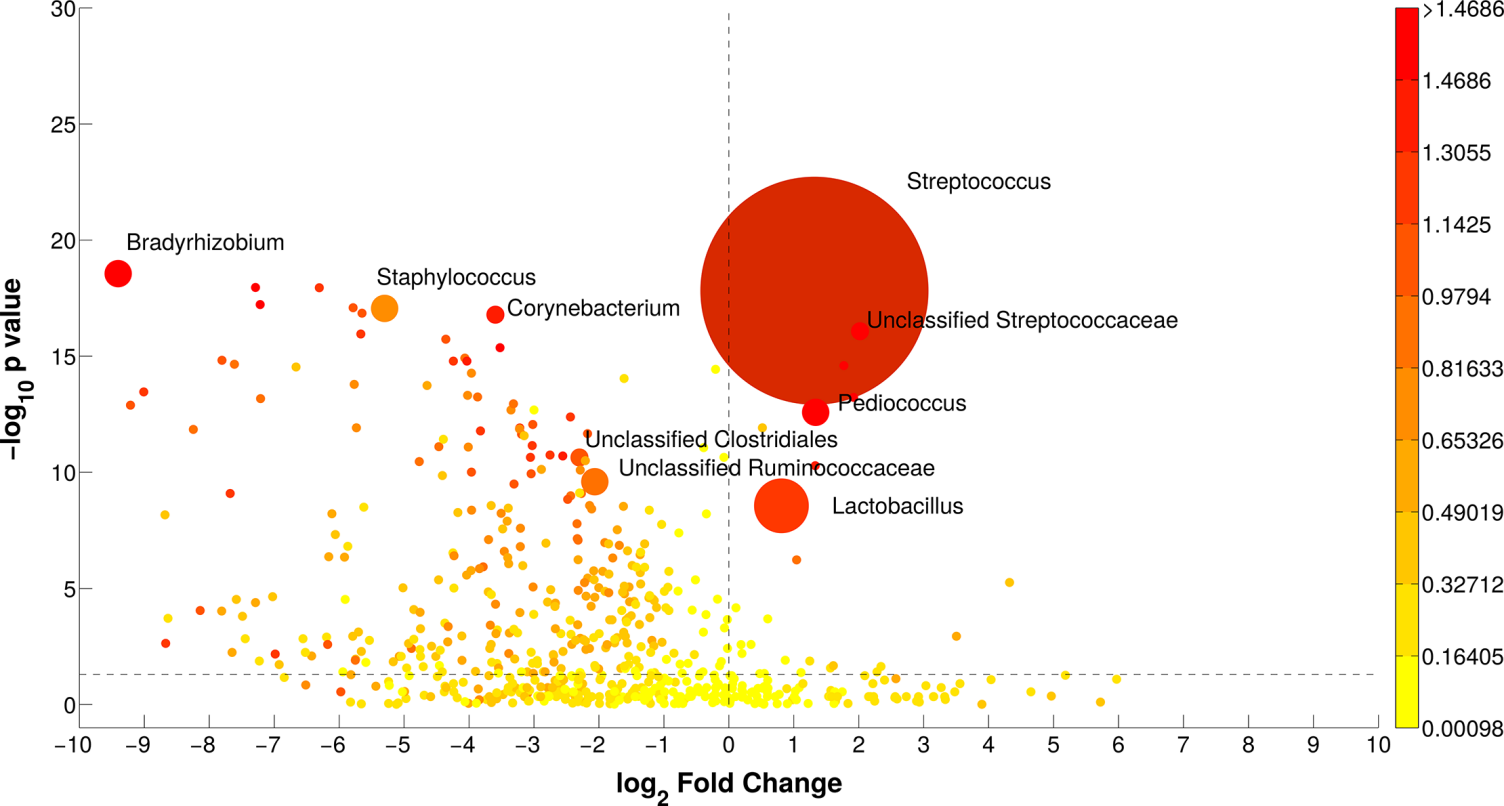
Bubble graph illustrating the groups significantly different between the two breeds (HF and REN) at genus level. X-axis reports the log_2_ ratio (REN/HF) of relative abundances; Y-axis depicts the–log_10_(p-value) of the two-sided Mann-Whitney U-test for comparing bacterial groups; bubble dimension is related to the average relative abundance of sequences; color code is according to Cohen's size effect. Bacterial groups namely indicated are the ones with relative abundance > 1%, p-value < 0.05 and log_2_(ratio) > 1.5.

At the family level (Fig 2B), the microbiota of the two breeds was characterized by significant differences in the average abundance of *Streptococcaceae* (HF 29.3%, REN 74.1%) and *Lactobacillaceae* (HF 6.9%, REN 14.0%). Significant differences were observed also for *Ruminococcaceae*, *Bradyrhizobiaceae*, *Aerococcaceae* and *Staphylococcaceae*, which were found almost exclusively in HF milk. At the genus level, both breeds were dominated by *Streptococcus*, although in very different proportions (average HF 27.5%, REN 68.6%); *Bradyrhizobium*, *Staphylococcus* and *Corynebacterium* were basically only present in HF milk, while *Lactobacillus* and *Pediococcus* were more present in REN milk. All these bacterial genera were diversely present in HF and REN milk (p-values < 0.05, Fig 3). A complete list of the bacterial groups at the phylum, family and genus levels, as well as their relative abundances in HF and REN milk, can be found in S2 Table.

HF and REN milk samples did also show a different core OTU composition. Considering the OTUs present in 100% of samples, the two breeds shared only two genera in their core microbiota: *Lactobacillus* and *Streptococcus*. The HF core was composed, alphabetically, by the genera *Bradyrhizobium*, *Corynebacterium*, *Lactobacillus*, *Propionibacterium*, *SMB53*, *Staphylococcus*, *Streptococcus*; the REN core, on the other hand, was composed by the genera *Enterococcus*, *Lactobacillus*, *Lactococcus*, *Leuconostoc*, *Pediococcus*, *Streptococcus*. The species-level analysis of sequences within the *Streptococcus* genus revealed that the main species in both breeds was likely *Str*. *thermophilus*. A minor quantity of environmental *Str*. *uberis* and *Str*. *dysgalactiae*, accounting for about 5–10% of the total relative abundance, was found only in a minority of HF samples (7 and 2 for *Str*. *uberis* and *Str*. *dysgalactiae*, respectively), as well as *Str*. *suis* in REN samples (present at about 0.6% of the total relative abundance in 20 REN samples) (S2 Fig).

### Comparison among time points

The second aim of this study was to assess the longitudinal changes occurring in the milk microbiota at the different time points (T1, T2, T3, T4) for both breeds. This was done by grouping the data from all quarters, separating the two breeds.

**Fig 4:**
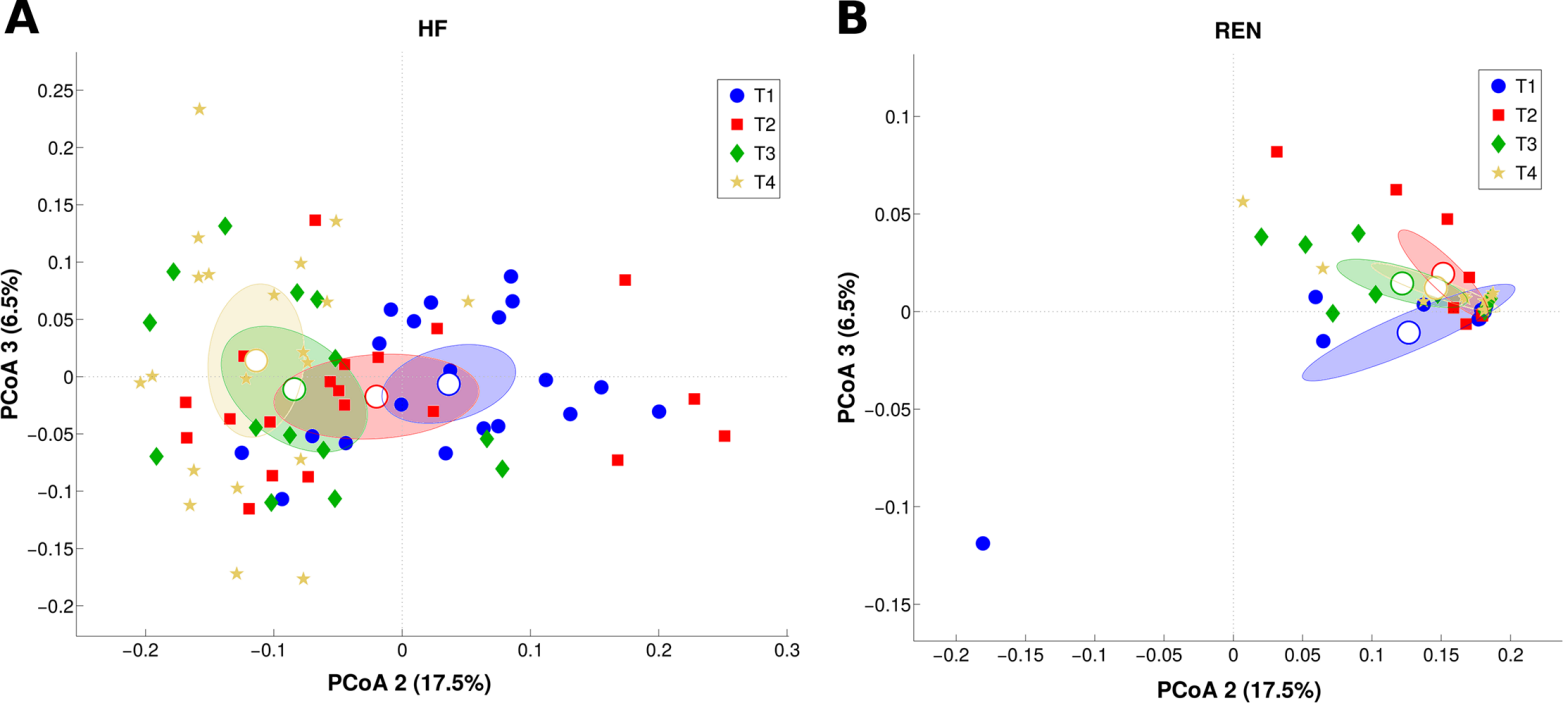
PCoA of weighted UniFrac distances representing the differences in milk microbiota structure along time points. Each dot represents a single quarter milk sample, while the centroid represents its average value. Percent variance accounted for by the second and third principal component is shown along the axis. **(A)** PCoA plots for HF; P-values are statistically significant (p < 0.02) for all pairwise comparison, except T2 *vs*. T3. **(B)** PCoA plots for REN; P-values are not statistically significant (p > 0.05).

According to our findings, the microbiota profile of the two breeds remained well separated at all time points, showing a high statistical significance (p < 0.001) on both weighted and unweighted Unifrac distances (S3 Fig). Fig 4 reports the differences between each time point in HF milk through the PCoA distribution; apart from T2 and T3, which were not statistically different, all other time points in HF showed a significant separation (p<0.05) on both Unifrac distances. On the other hand, the REN milk microbiota resulted indistinctly clustered at all time points. These results indicate that the microbial structure of HF milk changed profoundly throughout the calving period, while REN milk maintained a more stable microbiota composition.

We further investigated these differences by looking at the bacterial relative abundances for each time point per breed. At the genus level, the HF and REN milk microbiota composition varied in peculiar ways through time. In HF milk, an increase in *Streptococcus* (from 27.1% at T1 to 32.3% in T2), *Lactobacillus* (from 3.8% at T1 to 4.8% at T2) and *Bradyrhizobium* (from 1.7% at T1 to 4.7% at T2) was observed near the calving period, followed by a decrease at T4 back to dry-off values (S3 Table). In REN milk, *Streptococcus*, *Lactobacillus* and *Pediococcus* underwent a slight decrease right before and after calving but recovered at T4 (S4 Table).

### Somatic cell count and taxonomic composition

By comparing the SCC for the selected, healthy, quarter milk samples, higher values were seen in both breeds at the calving time point T2. Table 1 and S4 Fig report the correlations between bacterial taxa and SCC at different phylogenetic levels (p-value of the linear model < 0.01). Notably, we found a weak positive correlation between SCC and many bacterial groups belonging to the *Proteobacteria* phylum, such as those within the families of *Enterobacteriaceae*, *Sphingomonadaceae*, *Xanthomonadaceae* and *Pseudomonadaceae*.

**Table 1:** Correlation coefficients between REN and HF microbiota and SCC across all samples. Pearson’s linear correlation coefficient was calculated between SCC and relative abundances of microbial taxa on the 117 samples. Only correlations with a p-value of the linear model < 0.01 are reported. For each significant correlation, the average relative abundances of the specific taxa in REN and HF are reported.

		Avg. rel. ab (%)		Correlation coefficient
		REN	HF	
**phylum**	*Proteobacteria*	1.43	13.21	0.264
	Unclassified Bacteria	0.03	0.27	0.429
**class**	*Gammaproteobacteria*	0.77	3.41	0.335
	*Solibacteres*	0.00	0.33	0.265
	*Proteobacteria* (other)	0.03	0.28	0.360
	Unclassified Bacteria	0.03	0.27	0.429
**order**	*Enterobacteriales*	0.02	0.82	0.367
	*Sphingomonadales*	0.02	0.78	0.319
	*Xanthomonadales*	0.07	0.52	0.258
	*Solibacterales*	0.00	0.33	0.265
	*Proteobacteria* (other)	0.03	0.28	0.360
	Unclassified Bacteria	0.03	0.27	0.429
**family**	*Enterobacteriaceae*	0.02	0.82	0.367
	*Sphingomonadaceae*	0.02	0.77	0.321
	*Pseudomonadaceae*	0.09	0.43	0.266
	*Xanthomonadaceae*	0.07	0.40	0.288
	*Proteobacteria* (other)	0.03	0.28	0.360
	Unclassified Bacteria	0.03	0.27	0.429
**genus**	*Lactococcus*	1.30	0.52	0.250
	*Escherichia*	0.01	0.65	0.349
	*Novosphingobium*	0.01	0.48	0.270
	*Unclassified Solibacteriales*	0.00	0.32	0.278
	*Proteobacteria* (other)	0.03	0.28	0.360
	Unclassified Bacteria	0.03	0.27	0.429
	*Pseudomonas*	0.02	0.28	0.323

### Predictive metabolic analysis

**Fig 5:**
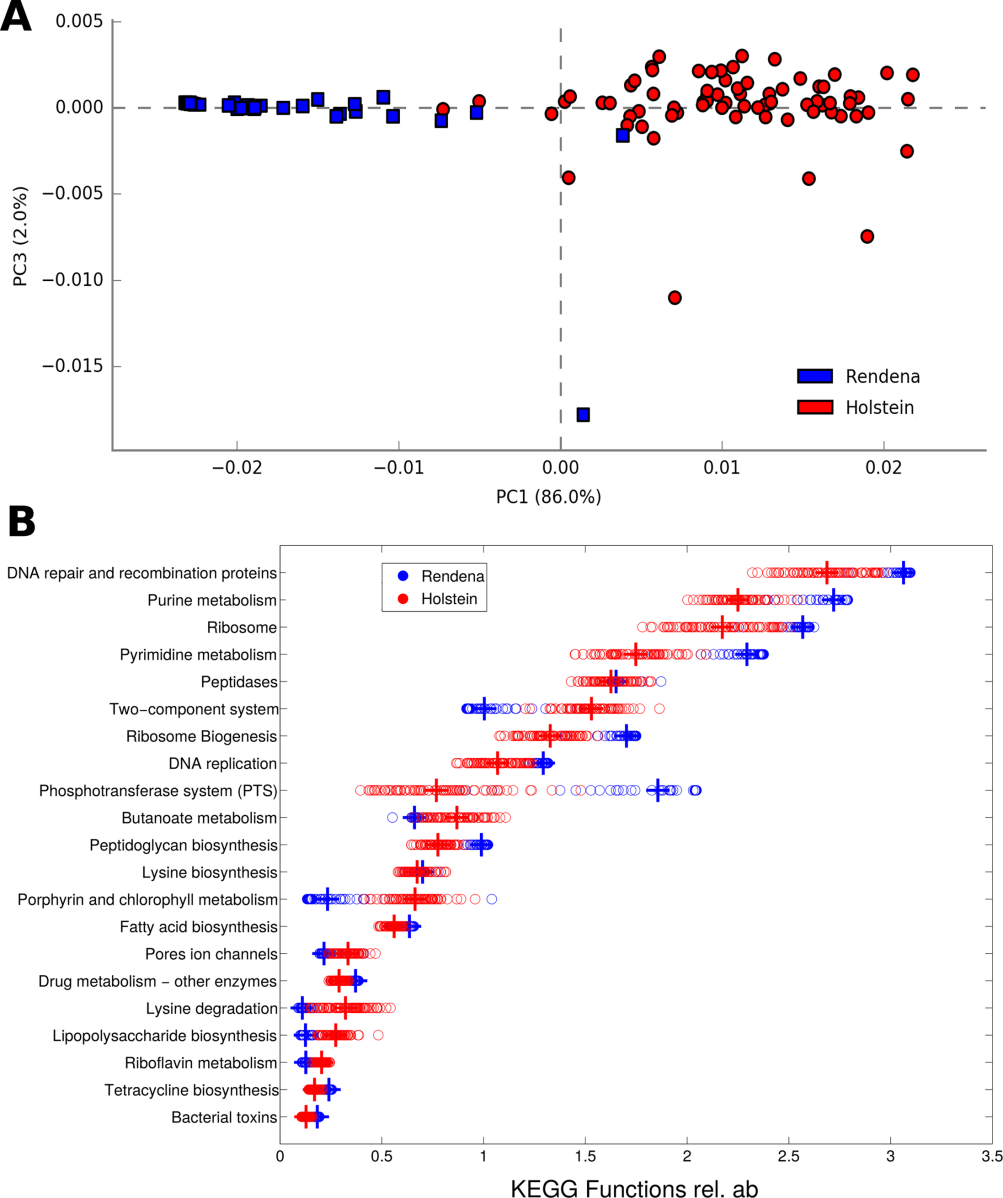
Functional comparison among HF and REN milk microbiota. **(A)** PCA of HF (red) and REN (blue) samples based on level 3 KEGG predicted pathways; the difference between breeds is highly significant (p = 0.001). Each dot represents a single quarter milk sample. Percent variance accounted for by the first and third principal component is shown. **(B)** Dot plot showing the specific level 3 KEGG predicted pathways that are enriched in REN and HF milk quarter samples. Most abundant gene categories for each breed were sorted out and the ratio between their averages was calculated. Only the first 20 significantly different gene categories between cow breeds (p-value < 0.05) are shown.

For each breed, 329 bacterial metabolic KEGG pathways at level 3, 41 at level 2, and 6,909 KO genes were analyzed. The predictive metabolic pathways at each level showed pronounced and significant differences (p = 0.001) in HF and REN milk concerning functional characterization (Fig 5A). Indeed, the two breeds had the same predicted functional composition, but in significantly different proportions. P-values were significant for the clear majority (i.e.: 272 out of 301, 90.4%) of the pathways suggesting that different microbial metabolic functions might be present in the milk of the two breeds, contributing to their peculiar characterization. Metabolic pathways such as butanoate metabolism and lipopolysaccharide biosynthesis were more present in HF milk microbiota, whereas cellular pathways like purine and pyrimidine metabolism, along with DNA proteins for repair and recombination and ribosomal proteins, were more present in REN milk microbiota. The main level 3 KEGG pathways are shown in Fig 5B.

## Discussion

The development of the so-called “-omics” technologies and progress in culture-independent techniques have strengthened the previous knowledge that milk is not sterile but harbors a diverse and complex microbial community [1, 33]. The selective pressure on HF cows based on production performances has led to their higher propensity to develop diseases in the transition period, including mastitis, and, perhaps, a different ability of the immune system to react against the environmental pressure [7, 16]. Conversely, less selected breeds, such as REN, are typically characterized by a lower milk production but a higher resistance to disease [20]. All this considered, to assess if structural differences in their microbial ecosystems might exist and if these might be related to mammary gland health, in this study we characterized the bovine mammary gland microbiota in healthy quarters of HF and REN in the transition period, when cows are more prone to develop disease [20, 34]. On the study farm, all cows were kept under the same conditions, so the influence of confounding factors such as diet, environment and animal management were minimal. This farming style created ideal conditions for a study aimed at understanding the reciprocal differences in the microbial composition of milk between the two breeds and during the transition period.

The milk microbiota has been found to vary among herds and geographical areas [35]. In our study, it was also shown to be significantly different both between breeds and during the calving period. Many more HF quarters compared to REN breed (i.e.: 13 *vs*. 6) were found to be contaminated during the experimental period, highlighting both an easier destabilization of the mammary gland microbiota and a lower defensive ability in HF during the periparturient period.

Consistent with the results of Falentin and co-workers [36], the taxonomic profiles of both HF and REN milk were dominated by Firmicutes, followed by Proteobacteria, Bacteroidetes, and Actinobacteria. A significantly lower diversity was observed in the microbial profile of REN milk for all the time points analyzed. At the genus level, only *Lactobacillus* and *Streptococcus* were shared between the two breeds, with *Streptococcus* being the most prevalent in both cases. Similar results were obtained during a study on milk samples derived from clinically healthy quarters [37].

The observed discrepancies between the two microbiota could bear on disease resistance in the mammary gland, in agreement with recent data about lactic acid bacteria [38]. Further investigations will be necessary to evaluate the real effect of some HF and REN bacteria on cow mammary gland diseases. In our study, the main species within the *Streptococcus* genus was *Str*. *thermophilus*, a lactic-acid bacterium widely used in the fermentation of dairy products (fermented milks, yogurt, different cheeses), which was present in both HF and REN milk, although in different proportions: it accounted for over 95% of the total *Streptococcus* abundance in REN, while it was less than 90% in HF. Species-level characterization will need further and more precise investigations to be confirmed, considering the debate concerning the possibility of obtaining species level identification based on V3-V4 regions of 16S rRNA, and the known difficulties in discriminating species within certain genera [39]. The differences in the microbial profile coincided, temporally, with the beginning of the lactation period, when metabolic and adaptation differences were observed between the two breeds. As previously reported [16, 20], it is worth underlining that HF showed both an increase in beta hydroxybutyrate (BOHB), responsible for immune functions depression, and more intense inflammatory phenomena. This situation can justify different responses even at a local level, as, for example, in the mammary gland [40].

There is increasing evidence in a variety of mammalian species that co-evolution of the microbiota with the innate immune system has resulted in elaborate interdependency and feedback mechanisms by which both systems control the mutual development and maintenance of host–microbe homeostasis [41–42]. In fact, the microbiota and the immune system are involved in a complex crosstalk that is influenced by innumerable environmental cues, and they interact both locally and across great distances within the body.

The different relative abundance at which every bacterial group was present in the two breeds suggested that the proportion of genes encoding each function might be different, possibly reflecting different metabolic activities. The imputed relative abundances of KEGG pathways were used to predict bacterial metabolic functions encoded by the milk microbiota of the two breeds (as in [43–44]), showing profound and significant differences between HF and REN. It is intriguing that REN’s milk major pathways seem to be more related to cellular processes at several levels (such as DNA proteins, nucleotides, ribosomes, phosphotransferase) while HF's relate to nutrients and cofactors (such as butanoate, riboflavin and lipopolysaccharide metabolisms; porphyrin and chlorophyll metabolism, belonging to “metabolism of cofactors and vitamins” KEGG category) and to two-component signal transduction systems, which enable bacteria to sense, respond, and adapt to environment or intracellular state changes [45]. These functional differences among breeds might provide a clue for further investigations on the mammary gland health.

Mechanisms such as nutrient competition, bacteriocins and antimicrobial molecules released by specific members of the bacterial community in milk may play a role in repressing the blooming of potential pathogens preventing intramammary infections. This was reported in a previous study on women investigating the role of the milk microbiota in intramammary infections and mastitis [46]. A role of the composition of milk microbiota in determining whether or not women would be affected by mastitis, as well as a host-microbiota dependence, has been suggested [47]. All this considered, bovine milk bacteria may also be crucial for programming the appropriate functionality of the immune system against pathogens and commensal bacteria.

Based on previous findings and on the protective role of a balanced microbiota, resistance to infections in the mammary gland might show breed-specific differences [1]. Interestingly, we found a positive correlation between increasing SCC and the relative abundance of bacterial opportunists, such as those belonging to the Proteobacteria phylum, which were found in higher amounts in HF milk microbiota, consistent with the higher incidence of mastitis and other transition-associated diseases in this breed [20]. It is worth noting that milk microbial profiles changed significantly along the transition period only in HF, while REN maintained a more stable microbiota composition.

## Conclusions

In this study, the implementation of high-throughput technologies for milk analysis provided detailed insights into the milk microbial population of a cosmopolitan breed, HF, and of a local cattle breed, REN, along the transition period. Our results highlighted the existence of differences in terms of general microbial diversity, taxonomy, and predicted functional profiles. In addition to the influence on the final characteristics of dairy products obtained from milk of the two breeds, those differences might also have an impact on their mammary gland health concerning disease and pathogen resistance. Interestingly, these differences seem related with inflammo-metabolic changes occurring around calving, which suggest a possible relation among these responses and the mechanisms of resistance in the mammary gland. Further studies carried out on a larger number of animals from both breeds will contribute to reinforce our findings in terms of inter-breed differences, the study of interactions between the microbiota and innate mechanisms of host defense, as well as the discrimination at and below the genus level.

## Supporting information

S1 Table Composition of the dry-off and lactating diets for both HF and REN cows. (PDF)

S2 Table Taxonomic characterization of milk microbiota differences among breeds at phylum, family and genus level. List of the bacterial groups with relative abundance > 1% and p-value < 0.05. Average relative abundance per breed (with related standard deviation), as well as Mann-Whitney U-test p-values, are reported.
(PDF)

S3 Table Genus level composition along the four time points for HF milk samples. Relative abundances (with related standard deviation) of the main bacterial groups along the four time points of sampling. On the right, the significance of the Mann-Whitney U-test is reported for each pair-wise comparison. P-values: ***: < 0.005; **: < 0.01; *: < 0.05.
(PDF)

S4 Table Genus level composition along the four time points for REN milk samples. Relative abundances (with related standard deviation) of the main bacterial groups along the four time points of sampling. On the right, the significance of the Mann-Whitney U-test is reported for each pair-wise comparison. P-values: ***: < 0.005; **: < 0.01; *: < 0.05.
(PDF)

S1 Fig Microbiota composition of outlier samples. The bacterial abundances at genus level are shown for the 8 discarded samples, as well as the average composition for the remaining REN (n = 43) and HF (n = 74) samples. **(A)** Relative abundances of the main commensals (green box) and environmental/opportunistic (red box) genera are shown as stacked barplot; **(B)** Main microbiota and environmental/opportunistic bacterial genera were grouped together and represented as stacked barplot, highlighting how the discarded samples had at ≥ 35% of environmental/opportunistic genera, compared to an average of ≤ 7% in other REN and HF samples.
(TIFF)

S2 Fig Species-level analysis of *Streptococcus*. The relative abundances of genus *Streptococcus* is shown for each quarter milk sample in the stacked bar plot **(A)** and in the pie charts **(B)**. The “Other” category (gray) represents all of the genera that do not belong to the genus *Streptococcus*; blue bars show how *Str*. *thermophilus* is the main species among the *Streptococcus* genus.
(TIFF)

S3 Fig PCoA of weighted UniFrac distances represent the differences in milk microbiota structure between each time point for HF and REN. Average distance between breeds is statistically significant (p = 0.01) for T1 **(A)**, T2 **(B)**, T3 **(C)**, T4 **(D)** time points.
(TIFF)

S4 Fig Correlation plots of SCC vs. microbial relative abundance. Dotplots represent the Pearson’s correlation coefficient between SCC and the relative abundance of selected bacterial taxa at different levels for all quarter milk samples (REN and HF). For representation purposes, SCC was log-transformed before plotting; only correlations with a p-value of the linear model < 0.01 are represented.
(TIFF)
