# Supplementary material for: Milk microbiome diversity and bacterial group prevalence in a comparison between healthy Holstein Friesian and Rendena cows
Source: PLoS One. 2018 Oct 24;13(10):e0205054. doi: 10.1371/journal.pone.0205054 (PMC6200206; doi:10.1371/journal.pone.0205054)
Supplement: S1 Table — (PDF) [file pone.0205054.s001.PDF]

	Diets			
	**Dry-off (Kg/cow)**	**DM**	**Lactating period (Kg/cow)**	**DM**
Grass hay	4	3.48	7	6.09
Alfalfa hay			5	4.35
Barley straw	6.5	5.72	2	1.76
Corn meal	1	0.88	6	5.28
Beet pulp	0.5	0.45	2	1.8
Sunflower Meal 24%	0.3	0.27	0.6	0.54
Flax	0.1	0.09	0.9	0.81
Vitamin mineral supplement	0.15	0.15	0.5	0.5
Total		11.04		21.13
