# Supplementary material for: Milk microbiome diversity and bacterial group prevalence in a comparison between healthy Holstein Friesian and Rendena cows
Source: PLoS One. 2018 Oct 24;13(10):e0205054. doi: 10.1371/journal.pone.0205054 (PMC6200206; doi:10.1371/journal.pone.0205054)
Supplement: S2 Table — List of the bacterial groups with relative abundance > 1% and p-value < 0.05. Average relative abundance per breed (with related standard deviation), as well as Mann-Whitney U-test p-values, are reported. (PDF) [file pone.0205054.s002.PDF]

Phylum	Average (StDev)		P-value
	Rendena	Holstein Friesian	
Firmicutes	94.6 (7.7)	66.6 (9.3)	6.79E-18
Proteobacteria	1.4 (2.1)	13.1 (6.8)	1.11E-17
Bacteroidetes	1.6 (3.9)	8.2 (4.5)	1.93E-15
Actinobacteria	0.9 (1.2)	6.7 (3.4)	8.27E-18
Planctomycetes	0.0 (0.1)	1.0 (0.8)	4.36E-16

Family	Average (StDev)		P-value
	Rendena	Holstein Friesian	
Streptococcaceae	73.9 (9.8)	29.1 (13.8)	1.52E-18
Lactobacillaceae	13.9 (3.5)	6.8 (4.5)	3.63E-12
Ruminococcaceae	1.2 (2.9)	5.7 (4.9)	1.34E-11
Bradyrhizobiaceae	0.1 (0.0)	4.8 (3.6)	3.42E-19
Aerococcaceae	0.6 (1.3)	4.1 (4.4)	2.80E-13
Staphylococcaceae	0.1 (0.3)	3.9 (5.8)	5.51E-17
Lachnospiraceae	0.3 (0.5)	3.3 (2.7)	1.35E-14
Leuconostocaceae	1.4 (0.5)	2.2 (2.6)	8.28E-01
Corynebacteriaceae	0.2 (0.4)	2.8 (2.2)	1.63E-17
Veillonellaceae	0.4 (0.8)	1.9 (2.1)	6.89E-09
Clostridiaceae	0.3 (0.4)	2.0 (1.2)	1.17E-15
Bacteroidaceae	0.3 (0.8)	1.9 (1.9)	6.63E-10
Moraxellaceae	0.5 (1.0)	1.4 (1.6)	1.79E-07
Unclassified Clostridiales	0.3 (0.6)	1.6 (1.5)	2.31E-11
Propionibacteriaceae	0.3 (0.5)	1.2 (0.9)	7.44E-13
Chitinophagaceae	0.0 (0.0)	1.4 (1.3)	1.13E-16

Genus	Average (StDev)		P-value
	Rendena	Holstein Friesian	
Streptococcus	68.6 (8.3)	27.5 (13.1)	1.52E-18
Lactobacillus	7.9 (2.0)	4.5 (3.4)	2.77E-09
Pediococcus	4.5 (1.2)	1.8 (1.5)	2.62E-13
Unclassified Streptococcaceae	2.9 (1.1)	0.7 (0.6)	8.47E-17
Lactobacillaceae (other)	1.4 (0.5)	0.4 (0.3)	2.54E-12
Leuconostoc	1.3 (0.5)	0.5 (0.7)	5.15E-11
Lactococcus	1.3 (0.4)	0.5 (0.6)	2.41E-13
Streptococcaceae (other)	1.0 (0.5)	0.3 (0.2)	5.89E-14
Unclassified Ruminococcaceae	0.9 (2.3)	3.8 (3.7)	2.53E-10
Unclassified Aerococcaceae	0.4 (1.1)	1.1 (1.5)	1.20E-07
Phascolarctobacterium	0.3 (0.6)	1.0 (1.5)	1.70E-05
Unclassified Clostridiales	0.3 (0.6)	1.6 (1.5)	2.30E-11
Propionibacterium	0.3 (0.5)	1.1 (0.9)	2.15E-12
Corynebacterium	0.2 (0.4)	2.9 (2.2)	1.63E-17
Unclassified Lachnospiraceae	0.2 (0.3)	1.2 (1.1)	7.01E-12
Staphylococcus	0.1 (0.2)	3.7 (5.8)	8.68E-18
SMB53	0.1 (0.2)	1.0 (0.7)	4.29E-16
Aerococcus	0.1 (0.2)	1.4 (2.3)	5.34E-15
Facklamia	0.1 (0.2)	1.3 (1.6)	1.21E-15
Weissella	0.0 (0.1)	1.2 (2.0)	1.63E-14
Bradyrhizobium	0.0 (0.0)	4.2 (3.4)	2.80E-19
Sediminibacterium	0.0 (0.0)	1.3 (1.3)	2.39E-17
