# Supplementary material for: Milk microbiome diversity and bacterial group prevalence in a comparison between healthy Holstein Friesian and Rendena cows
Source: PLoS One. 2018 Oct 24;13(10):e0205054. doi: 10.1371/journal.pone.0205054 (PMC6200206; doi:10.1371/journal.pone.0205054)
Supplement: S3 Table — Relative abundances (with related standard deviation) of the main bacterial groups along the four time points of sampling. On the right, the significance of the Mann-Whitney U-test is reported for each pair-wise comparison. P-values: ***: < 0.005; **: < 0.01; *: < 0.05. (PDF) [file pone.0205054.s003.PDF]

	Average (StDev)				p-value			
	T1	T2	T3	T4		vs T2	vs T3	vs T4
					T1			*
Streptococcus	27.1 (13.6)	32.3 (13.6)	27.7 (10.7)	23.1 (12.9)	T2	-		
					T3	-	-	
					T1			
Lactobacillus	3.8 (2.3)	4.8 (2.7)	5.6 (5.3)	4.1 (3.2)	T2	-		
					T3	-	-	
					T1			
Pediococcus	2.3 (1.9)	2.1 (1.3)	1.3 (0.9)	1.4 (1.6)	T2	-		
					T3	-	-	
					T1		*	
Unclassified Streptococcaceae	0.9 (0.7)	0.7 (0.5)	0.7 (0.5)	0.5 (0.5)	T2	-		
					T3	-	-	
					T1			*
Lactobacillaceae (other)	0.5 (0.4)	0.5 (0.3)	0.4 (0.2)	0.3 (0.3)	T2	-		*
					T3	-	-	*
					T1			
Leuconostoc	0.5 (0.5)	0.6 (0.6)	0.5 (0.5)	0.5 (1.0)	T2	-		
					T3	-	-	
					T1			
Lactococcus	0.5 (0.4)	0.7 (1.0)	0.5 (0.4)	0.3 (0.4)	T2	-		
					T3	-	-	
					T1			
Streptococcaceae (other)	0.3 (0.2)	0.3 (0.2)	0.2 (0.1)	0.2 (0.3)	T2	-		
					T3	-	-	
					T1			*
Unclassified Ruminococcaceae	5.3 (3.9)	3.9 (4.6)	3.6 (3.3)	2.3 (2.0)	T2	-		
					T3	-	-	
					T1			
Unclassified Aerococcaceae	0.8 (0.6)	1.0 (1.6)	1.0 (1.0)	1.5 (2.1)	T2	-		
					T3	-	-	
					T1		*	*
Phascolarctobacterium	1.3 (1.0)	1.5 (2.4)	0.6 (0.6)	0.6 (0.9)	T2	-		
					T3	-	-	

	Average (StDev)				p-value			
	T1	T2	T3	T4		vs T2	vs T3	vs T4
Unclassified Clostridiales					T1			*
	1.9 (1.4)	1.8 (1.9)	1.2 (1.0)	1.3 (1.3)	T2	-		
					T3	-	-	
Propionibacterium					T1	*	***	
	0.7 (0.4)	1.5 (1.1)	1.5 (1.0)	0.9 (0.8)	T2	-		*
					T3	-	-	*
Corynebacterium					T1			
	2.5 (2.5)	2.5 (1.7)	3.5 (2.5)	3.1 (2.1)	T2	-		
					T3	-	-	
Unclassified Lachnospiraceae					T1			*
	1.6 (1.2)	1.2 (1.2)	1.0 (0.9)	0.8 (0.8)	T2	-		
					T3	-	-	
Staphylococcus					T1	***		
	6.0 (8.4)	1.0 (0.8)	2.1 (1.4)	5.4 (6.0)	T2	-	*	***
					T3	-	-	
SMB53					T1			
	0.9 (0.8)	0.8 (0.5)	1.0 (0.9)	1.3 (0.7)	T2	-		*
					T3	-	-	
Aerococcus					T1	*	*	
	1.5 (1.8)	1.0 (2.2)	0.7 (1.0)	2.0 (3.3)	T2	-		*
					T3	-	-	*
Facklamia					T1	*		**
	1.0 (0.8)	0.8 (1.7)	1.4 (1.9)	2.0 (1.7)	T2	-		***
					T3	-	-	
Weissella					T1			
	0.76 (0.9)	0.77 (1)	0.59 (0.69)	2.4 (3.4)	T2	-		
					T3	-	-	
Bradyrhizobium					T1	**	***	***
	1.7 (2.1)	4.7 (3.6)	5.1 (3.1)	5.5 (3.4)	T2	-		
					T3	-	-	
Sediminibacterium					T1	**	***	***
	0.6 (1.1)	0.9 (0.7)	1.8 (1.4)	2.1 (1.2)	T2	-	*	***
					T3	-	-	
