# Supplementary material for: Milk microbiome diversity and bacterial group prevalence in a comparison between healthy Holstein Friesian and Rendena cows
Source: PLoS One. 2018 Oct 24;13(10):e0205054. doi: 10.1371/journal.pone.0205054 (PMC6200206; doi:10.1371/journal.pone.0205054)
Supplement: S4 Table — Relative abundances (with related standard deviation) of the main bacterial groups along the four time points of sampling. On the right, the significance of the Mann-Whitney U-test is reported for each pair-wise comparison. P-values: ***: < 0.005; **: < 0.01; *: < 0.05. (PDF) [file pone.0205054.s004.PDF]

	Average (stdev)				p-value			
	T1	T2	T3	T4		vs T2	vs T3	vs T4
					T1		*	
Streptococcus	69.3 (12.1)	68.7 (7.8)	67.7 (5.5)	68.8 (6.6)	T2	-		
					T3	-	-	
					T1			*
Lactobacillus	7.6 (2.2)	7.3 (1.8)	7.8 (1.8)	9.0 (2.0)	T2	-		*
					T3	-	-	
					T1			
Pediococcus	4.5 (1.2)	4.1 (1.3)	4.6 (1.1)	5.0 (1.1)	T2	-		
					T3	-	-	
					T1			
Unclassified Streptococcaceae	3.1 (1.2)	2.7 (1.1)	2.9 (1.1)	2.9 (1.2)	T2	-		
					T3	-	-	
					T1			
Lactobacillaceae (other)	1.6 (0.5)	1.3 (0.5)	1.4 (0.6)	1.4 (0.5)	T2	-		
					T3	-	-	
					T1			
Leuconostoc	1.4 (0.4)	1.4 (0.4)	1.3 (0.6)	1.3 (0.6)	T2	-		
					T3	-	-	
					T1			
Lactococcus	1.3 (0.3)	1.3 (0.3)	1.2 (0.3)	1.4 (0.4)	T2	-		
					T3	-	-	
					T1			
Streptococcaceae (other)	1.1 (0.6)	0.9 (0.5)	1.0 (0.5)	1.0 (0.6)	T2	-		
					T3	-	-	
					T1			
Unclassified Ruminococcaceae	1.7 (4.1)	0.3 (0.5)	1.0 (1.1)	0.6 (1.0)	T2	-		
					T3	-	-	
					T1			
Unclassified Aerococcaceae	0.1 (0.2)	1.1 (2.0)	0.4 (0.5)	0.1 (0.2)	T2	-		
					T3	-	-	
					T1			
Phascolarctobacterium	0.3 (0.6)	0.2 (0.5)	0.5 (0.5)	0.4 (0.8)	T2	-		
					T3	-	-	

	Average (stdev)				p-value			
	T1	T2	T3	T4		vs T2	vs T3	vs T4
Unclassified Clostridiales	0.4 (0.7)	0.1 (0.3)	0.5 (0.6)	0.3 (0.6)	T1			
					T2	-		
					T3	-	-	
Propionibacterium	0.1 (0.1)	0.2 (0.2)	0.3 (0.2)	0.5 (0.8)	T1			*
					T2	-		
					T3	-	-	
Corynebacterium	0.1 (0.1)	0.3 (0.2)	0.5 (0.8)	0.1 (0.2)	T1			
					T2	-		
					T3	-	-	
Unclassified Lachnospiraceae	0.1 (0.3)	0.1 (0.1)	0.2 (0.3)	0.2 (0.3)	T1			
					T2	-		
					T3	-	-	
Staphylococcus	0.1 (0.0)	0.2 (0.3)	0.1 (0.2)	0.1 (0.2)	T1			
					T2	-		
					T3	-	-	
SMB53	0.0 (0.1)	0.1 (0.1)	0.2 (0.4)	0.0 (0.1)	T1			
					T2	-		
					T3	-	-	
Aerococcus	0.0 (0.0)	0.1 (0.1)	0.1 (0.2)	0.2 (0.4)	T1			
					T2	-		
					T3	-	-	
Facklamia	0.0 (0.0)	0.1 (0.1)	0.2 (0.3)	0.1 (0.0)	T1			
					T2	-		
					T3	-	-	
Weissella	0.0 (0.0)	0.0 (0.0)	0.1 (0.1)	0.0 (0.0)	T1			
					T2	-		
					T3	-	-	
Bradyrhizobium	0.0 (0.0)	0.0 (0.0)	0.0 (0.0)	0.0 (0.0)	T1			
					T2	-		
					T3	-	-	
Sediminibacterium	0.0 (0.0)	0.0 (0.0)	0.0 (0.0)	0.0 (0.0)	T1			
					T2	-		
					T3	-	-	
