# Supplementary figures and images for: Milk microbiome diversity and bacterial group prevalence in a comparison between healthy Holstein Friesian and Rendena cows
Source: PLoS One. 2018 Oct 24;13(10):e0205054. doi: 10.1371/journal.pone.0205054 (PMC6200206; doi:10.1371/journal.pone.0205054)

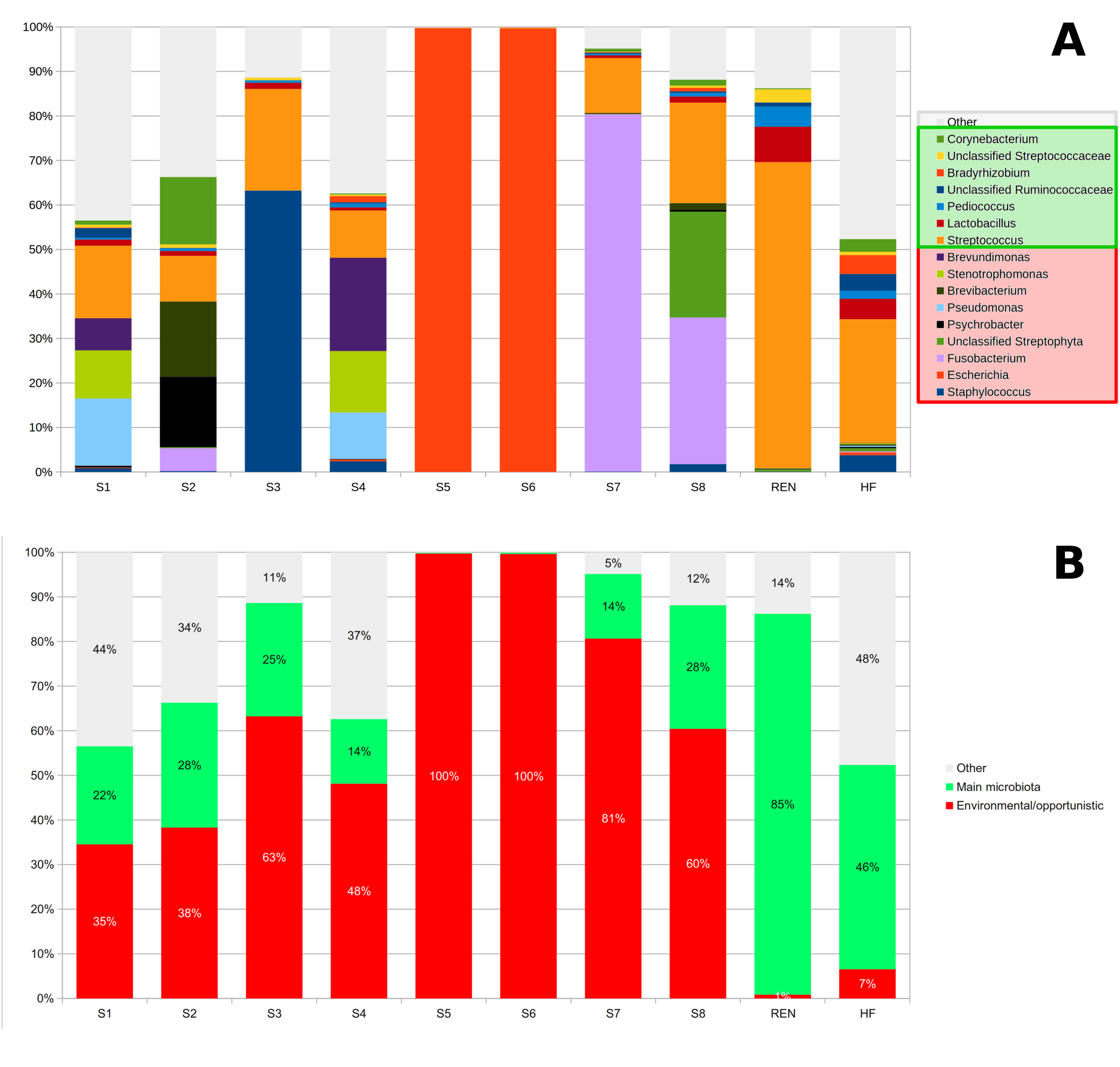

Supplement: S1 Fig — The bacterial abundances at genus level are shown for the 8 discarded samples, as well as the average composition for the remaining REN (n = 43) and HF (n = 74) samples. (A) Relative abundances of the main commensals (green box) and environmental/opportunistic (red box) genera are shown as stacked barplot; (B) Main microbiota and environmental/opportunistic bacterial genera were grouped together and represented as stacked barplot, highlighting how the discarded samples had at ≥ 35% of environmental/opportunistic genera, compared to an average of ≤ 7% in other REN and HF samples. (TIFF) [file pone.0205054.s005.tiff]

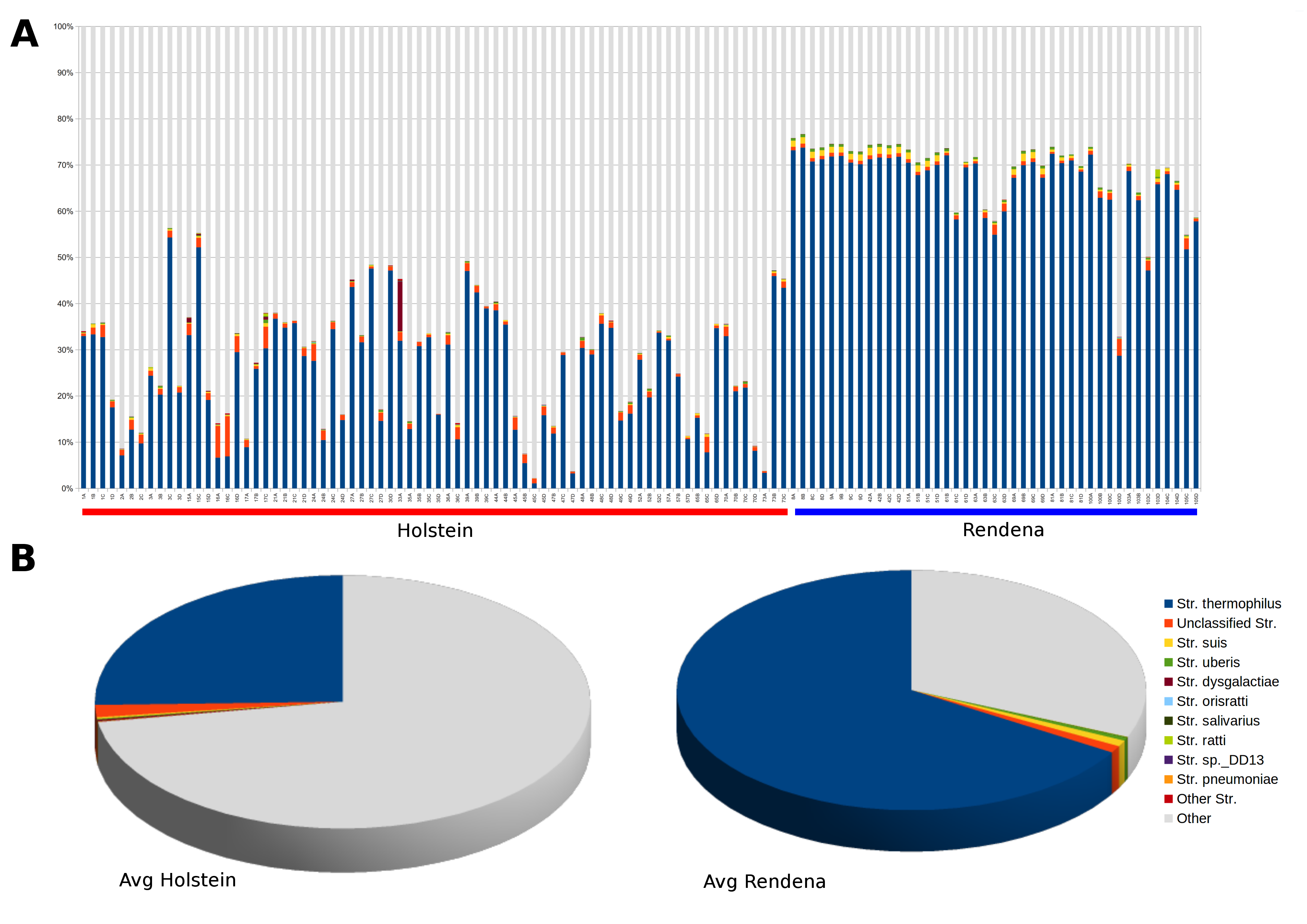

Supplement: S2 Fig — The relative abundances of genus Streptococcus is shown for each quarter milk sample in the stacked bar plot (A) and in the pie charts (B). The “Other” category (gray) represents all of the genera that do not belong to the genus Streptococcus; blue bars show how Str. thermophilus is the main species among the Streptococcus genus. (TIFF) [file pone.0205054.s006.tiff]

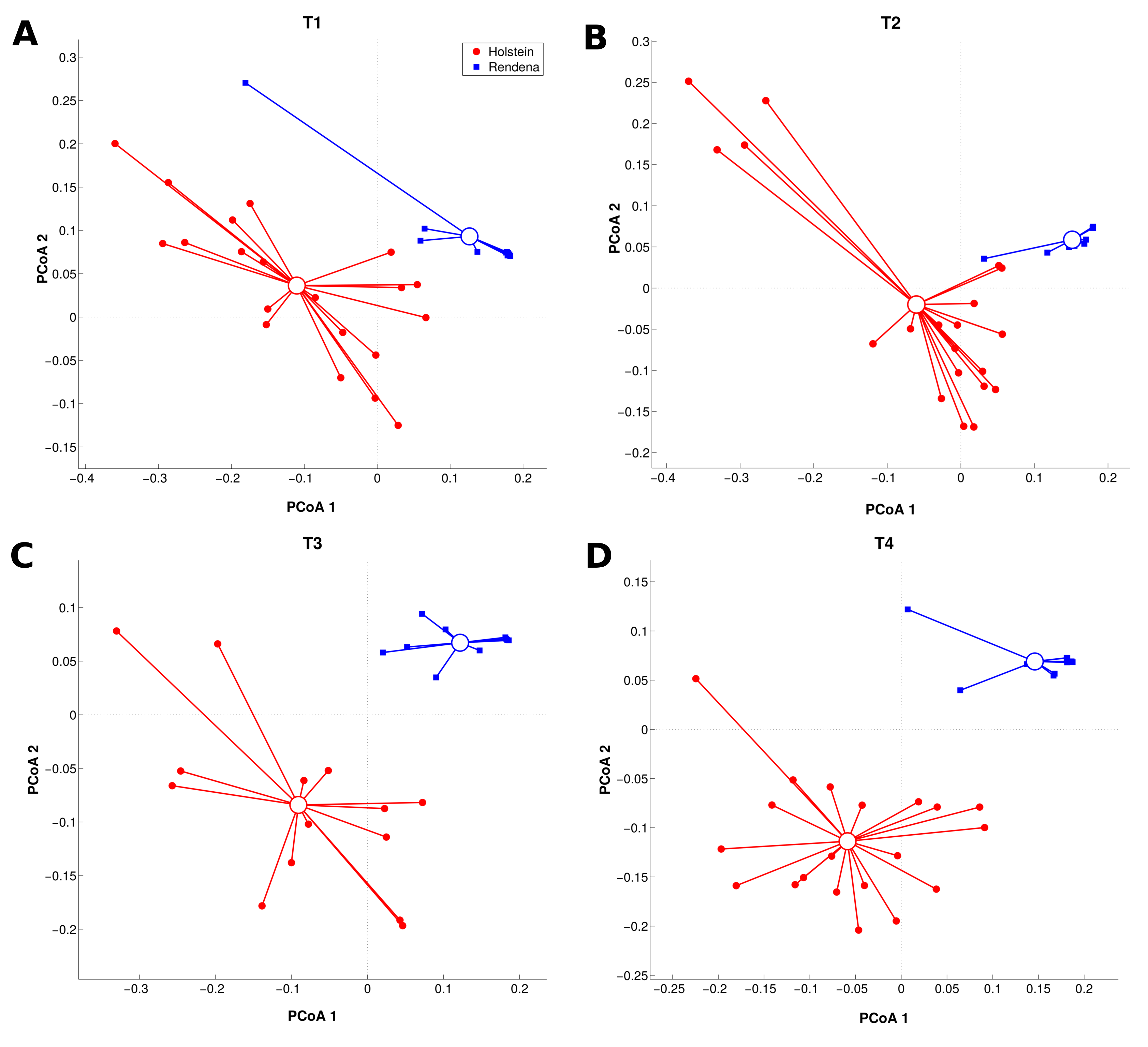

Supplement: S3 Fig — Average distance between breeds is statistically significant (p = 0.01) for T1 (A), T2 (B), T3 (C), T4 (D) time points. (TIFF) [file pone.0205054.s007.tiff]

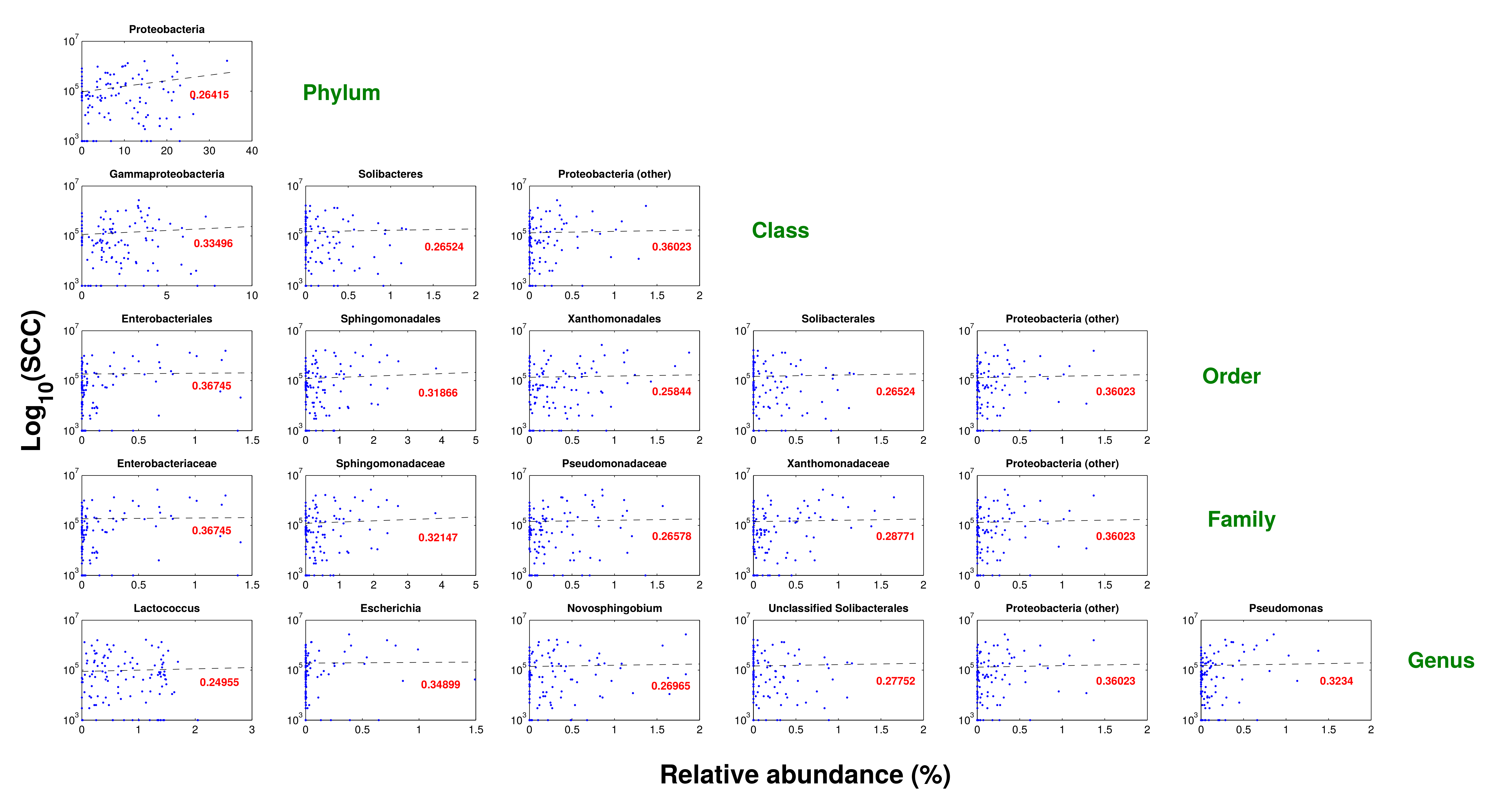

Supplement: S4 Fig — Dotplots represent the Pearson’s correlation coefficient between SCC and the relative abundance of selected bacterial taxa at different levels for all quarter milk samples (REN and HF). For representation purposes, SCC was log-transformed before plotting; only correlations with a p-value of the linear model < 0.01 are represented. (TIFF) [file pone.0205054.s008.tiff]
